# Ecological risk assessment of migratory bird feces in plateau wetland: Phosphorus speciation, ARGs, and pathogens

**DOI:** 10.1371/journal.pone.0348709

**Published:** 2026-09-21

**Authors:** Yunchuan Long, Jian Lang, Longyan Wang, Jin Guo, Xuankong Jiang, Juan Jiang

**Affiliations:** 1 Guizhou Key Laboratory of Plateau Wetland Conservation and Restoration, Guizhou Institute of Biology, Guizhou Academy of Sciences, Guiyang, China; 2 College of Resources and Environmental Engineering, Key Laboratory of Karst Georesources and Environment, Ministry of Education, Guizhou University, Guiyang, China; MARE – Marine and Environmental Sciences Centre, PORTUGAL

## Abstract

Plateau wetlands are critical habitats for migratory birds, but the potential ecological risks from the large-scale accumulation of migratory bird feces (MBF) remain poorly understood. This study focused on seven wintering waterbird species, including the *Grus nigricollis*, *Grus grus, Fulica atra, Anas strepera, Anas zonorhyncha, Mareca penelope,* and *Tadorna ferruginea* at Caohai Plateau Wetland, sampling across March 2024, January 2025 and March 2025*.* The phosphorus (P) speciation, antibiotic resistance genes (ARGs), pathogens, and their interactions were assessed using sequential phosphorus extraction, solution ^31^P-nuclear magnetic resonance (^31^P-NMR), and shotgun metagenomic sequencing. Results showed that MBF was enriched in nutrients and heavy metals, with Gruiformes feces containing high total P (0.65% dry weight). Orthophosphate accounted for 90.68% of total P and labile NaHCO_3_-P_o_ constituted the dominant organic P fraction, collectively indicating strong guanotrophication potential. Metagenomic annotation recovered 231 potential pathogens with *Staphylococcus aureus*, *Salmonella enterica*, and *Pseudomonas aeruginosa* as dominant taxa; 21 ARG classes were identified, dominated by multidrug resistance genes (32.14–39.29%). Heavy metal (Cu, Cd, Zn) selection pressure enriched MGEs in avian gut microbiota, thereby facilitating horizontal transfer of ARGs and virulence factors (VFs) and ultimately driving the enrichment of multi-drug resistant pathogens. The low-temperature plateau environment further extended the environmental persistence of these hazardous biological contaminants. This study provides a synergistic risk network linking nutrient loading, heavy metal pollution, antimicrobial resistance, and pathogenic proliferation in MBF-impacted plateau wetlands, providing scientific support for plateau wetland ecological restoration and targeted public health risk mitigation under the One Health framework.

## Introduction

The East Asian-Australasian Flyway, one of the nine major global flyways, extends from the Russian Far East and Alaska in the north to Australia and New Zealand in the south, and supports the migration of more than 50 million migratory waterbirds [1]. As unique but fragile ecosystems, plateau wetlands play irreplaceable ecological roles by providing critical stopover, breeding, and wintering habitats for migratory birds and support numerous waterfowl species [2,3]. Waterbirds provide some essential ecosystem services, including: provisioning services (meat, eggs, feathers, grease, etc.), supporting services (animal & plant dispersal, nutrient cycling, primary productivity, biodiversity, bioindicators, etc.), regulating services (pest control, disease surveillance, regime shifts, etc.), and recreational services (birdwatching, ecotourism, art, etc.) [4]. Despite its immense importance, the massive accumulation of migratory bird feces (MBF) in wetlands has gradually become a non-negligible ecological disturbance factor, posing potential risks to the structure and function of wetland ecosystems and even human health [5]. MBF are rich in nutrients [6,7], pathogens [8], and various functional genes [9], which can significantly alter the biogeochemical cycles of wetland environments and induce ecological risks.

The excretion of bird droppings introduces substantial external nutrients into habitats, profoundly altering nutrient cycling and thereby affecting primary productivity, ecological structure, and function of both aquatic and terrestrial ecosystems near bird colonies [10]. As a key limiting nutrient for primary productivity in wetlands [11], phosphorus (P) in bird guano exists in various chemical forms, with orthophosphate (ortho-P) being the most mobile and bioavailable [10]. Unlike conventional anthropogenic P sources (e.g., agricultural runoff, domestic sewage), P derived from MBF exhibits distinct characteristics, with high proportions of bioavailable phosphorus [6,12]. These labile forms are highly susceptible to rapid microbial mineralization and abiotic transformations [6], which can trigger “guanotrophication” (eutrophication from guano). Indeed, Adhurya and Ray [4] confirmed that waterbirds deposit sufficient nutrients to pose eutrophication risks, and Alba-González et al. [13] highlighted that phytoplankton-bacteria interactions regulate the influence of fecal nutrients on primary productivity. Nutrient levels and P speciation vary by bird species: for instance, Suliformes display higher levels of P with 15.08 ± 1.07%, while Procellariiformes exhibit higher N content at 19.13 ± 3.46% [14]. Despite such taxonomic variability, the biogeochemical characterization of P species in plateau bird feces from different species and the associated eutrophication risks remains critically underexplored.

Antimicrobial resistance has become a major global public health concern [15]. Among them, ARGs, as a class of emerging environmental pollutants, not only can proliferate and propagate between microbial communities but also pose a significant threat to public health [16,17]. Furthermore, ARGs can proliferate and disseminate among various bacterial genera through bacterial growth and horizontal gene transfer (HGT) via MGEs, such as plasmids, integrons, and insertion sequences [16,18]. Several studies have highlighted the role of wildlife as reservoirs and vectors of antibiotic-resistant bacteria and ARGs [19]. The apex predators, such as waterbirds, represent a reservoir for resistant bacteria, and their gut microbial communities provide opportunities for the exchange of ARGs between bacteria by HGT, adding dimension to the emergence and dissemination of resistance [20]. Furthermore, individuals from wildlife species that travel over extensive spatial distributions can also facilitate wide-range dispersal of resistant bacteria [15]. Bird feces often contain antibiotics and pathogenic bacteria, facilitating the survival of antibiotic-resistant bacteria [12]. Migratory birds, with their long-distance migration characteristics, can spread pathogens across different regions, breaking the geographical isolation of microbial communities [19,21]. Pathogens such as *Salmonella*, *Campylobacter*, and Avian Influenza Virus in MBF can enter wetland ecosystems through fecal deposition and then spread to humans and other animals via water contact, food chain transmission, or aerosolization [8]. Furthermore, the interactions between MBF-derived P, pathogens, and ARGs in plateau wetlands have not been reported, which hinders the comprehensive assessment of the ecological and health risks posed by MBF.

The rapid advancement of metagenomic sequencing technology has broken the limitations of traditional detection methods, providing a powerful tool for comprehensive exploration of the composition, distribution, and transmission mechanisms of resistant microorganisms and ARGs in complex environments [22]. A recent nationwide survey characterized gut resistomes from 52 bird species across 11 provinces in China [23]. However, no equivalent dataset exists for the Guizhou plateau wetland avifauna. Existing studies typically address guanotrophication [4,7], fecal microbiome composition [5,21,24], or ARG distribution [16,19,20] in isolation; remarkably few integrate nutrients, metal pressure, ARG reservoirs, and zoonotic pathogen assemblages within bird feces. This knowledge fragmentation impedes holistic risk assessment of coupled eutrophication and zoonotic disease emergence driven by avian guano, and obscures mechanistic links between geochemical drivers and the horizontal dissemination of antimicrobial resistance across wetland ecosystems. Given the unique ecological functions of plateau wetlands, there is an urgent need to conduct a systematic ecological risk assessment of MBF. The present study integrated the application of metagenomic and ^31^P-NMR techniques, targeting the fecal samples of migratory birds in plateau wetlands as the research subject, to analyze the ecological risks associated with P species, ARGs, and pathogens. It aims to (1) characterize the P species derived from MBF through chemical extraction and ^31^P-NMR methods; (2) investigate the diversity, abundance, ARGs, and potential pathogenicity of microorganisms in MBF; (3) evaluate the composite ecological risks of MBF from the perspectives of eutrophication, pathogen transmission, and ARGs dissemination. This study will provide a scientific basis for the ecological protection and management of plateau wetlands, as well as the prevention of public health risks associated with migratory birds.

## Materials and methods

### Sampling collection and processing

**Fig 1:**
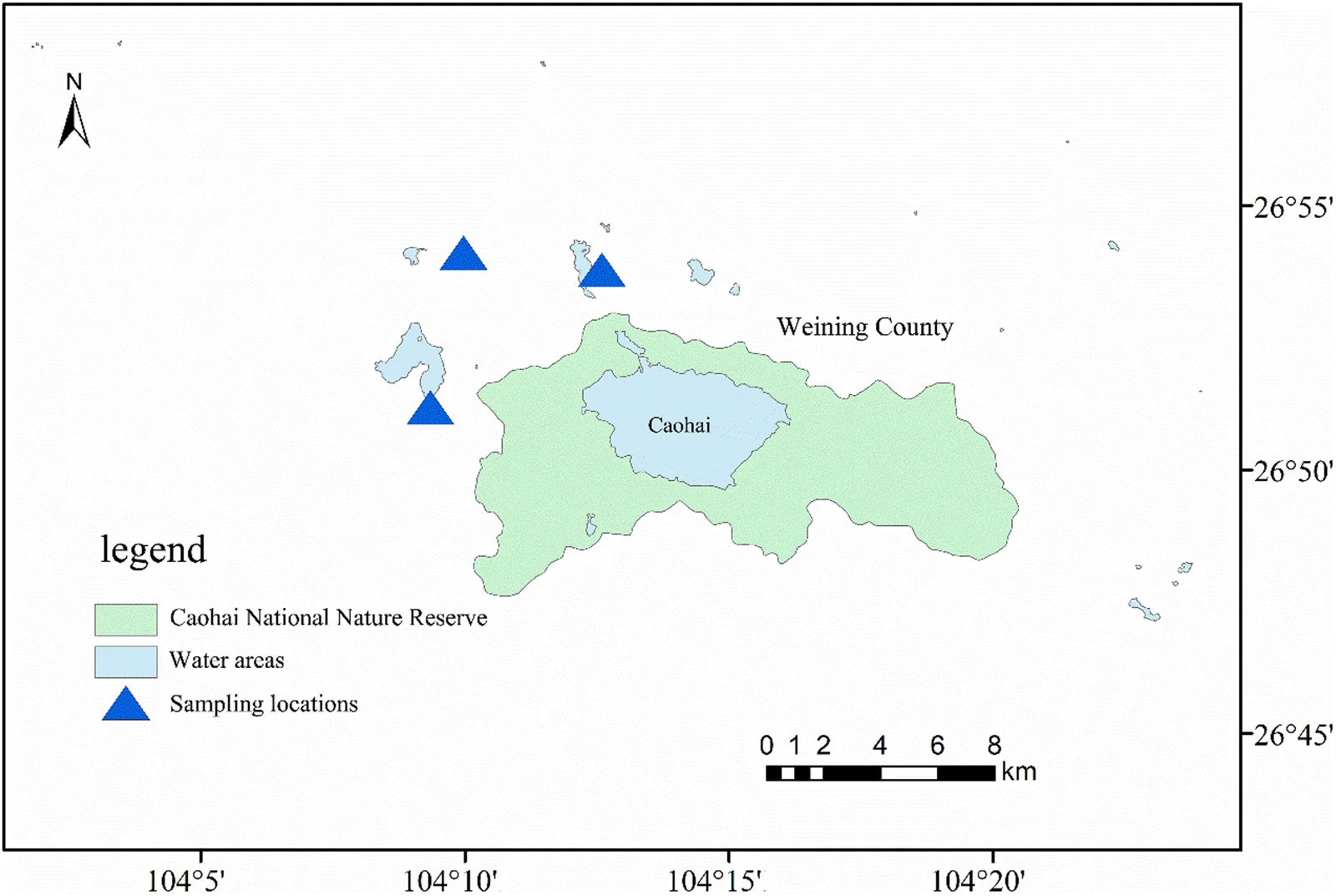
Sampling locations of bird feces in the Caohai Wetland.

The study was conducted in Caohai Wetland (Fig. 1), a representative and ecologically sensitive plateau freshwater wetland ecosystem, situated in the Wumeng Mountain hinterland of the central Yunnan-Guizhou Plateau (Guizhou Province, Southwest China) at an altitude of approximately 2200 m [25]. It covers a surface water area of 19.8 km^2^ and experiences a subtropical plateau monsoon climate [2]. Caohai Wetland functions as a key wintering habitat for migratory birds along the East Asia–Australasia Flyway, supporting over 285 avian species and hosting more than 100,000 migratory individuals annually, including 16 nationally first-class and 49 second-class protected species, of which 109 are waterbirds.

Fresh fecal samples were collected from seven migratory bird species: black-necked cranes (*Grus nigricollis*), *Grus grus, Fulica atra, Anas strepera, Anas zonorhyncha, Mareca penelope,* and *Tadorna ferruginea,* during March 2024, January 2025 and March 2025. During each sampling period, fecal samples were collected from all seven species whenever feasible. Taxonomically, *G. nigricollis, G. grus,* and *F. atra* belong to the order Gruiformes, while *A. strepera*, *A. zonorhyncha*, *M. penelope,* and *T. ferruginea* belong to the order Anseriformes. *G. nigricollis* represents a plateau-endangered species listed as Class I nationally protected wildlife in China, and *G. grus* is designated as Class II nationally protected wildlife. The remaining four anseriform species and *F. atra* are dominant waterfowl with stable large populations. Critically, all seven species exhibit typical long-distance migratory behaviours, rendering them ideal subjects for investigating the cross-regional dissemination of contaminants and pathogens.

Sampling was conducted opportunistically: birds were observed continuously, and fresh fecal samples were collected immediately upon defecation using sterile gloves [5]. For each species, fecal samples were collected at least 5 m, and more than 10 independent, fresh, solid fecal samples were obtained per species. The collected fecal samples were immediately placed into a cooling box with dry ice for transportation to the laboratory, where samples were vacuum freeze-dried, ground into powder (< 60 mesh) and then stored at −80 °C for subsequent analysis. C, H, N, and S were measured using an elemental analyzer (Elementar Unicube, Germany). The metal concentration was determined using an inductively coupled plasma emission spectrometer (ICP–OES, iCAP PRO, Thermo Fisher, USA).

### Ethics statement

All fresh avian faecal samples were collected from private farmlands within migratory bird foraging areas adjacent to Caohai National Nature Reserve, but outside the reserve’s protected zones. Prior to sampling, the research team informed local landholders of the study’s objectives, sampling locations and procedures, sample types, and downstream analytical uses, and obtained their verbal informed consent. As the sampling sites are conventional household agricultural land and collection involved no land-use alteration, property damage, or ecological disturbance, no special land access permit was required under local regulations. Moreover, because only naturally deposited faeces were collected—without capturing, restraining, or otherwise handling live birds—no wildlife handling permits were necessary. All procedures were conducted in strict compliance with ethical guidelines for wildlife research, relevant national and local regulations, and the principles of animal welfare and environmental protection.

### Phosphorus speciation analysis by chemical extraction and ^31^P-NMR

P speciation was analyzed using the Standards, Measurements and Testing Program (SMT) extraction scheme. Three fractions of P were classified as redox-sensitive P (Fe-P, extracted for 16 h with 1 M NaOH), strongly fixed P (Ca-P, extracted for 16 h with 1 M HCl), and organophosphorus (P_o_) [26]. The fractions of P_o_ in the fecal samples were measured using the Ivanoff sequential extraction protocol [11]. In brief, this method enables the fractionation of P_o_ into three fractions: labile P_o_, moderately labile P_o_ (composed of HCl-P_o_ and Ful-P_o_), and non-labile P_o_ (consisting of Hum-P_o_ and Res-P_o_). The concentrations of P in the extracts were determined using the molybdenum blue spectrophotometric method. All samples were processed in three independent replicates during P extraction. At each extraction step, two consecutive extractions were conducted under identical conditions, and the resulting supernatants were pooled quantitatively to ensure complete recovery of the target P fractions. Phosphorus speciation analysis was performed in analytical triplicate for each fraction, and the average values were used for data analysis.

To further deconstruct the P speciation in the bird feces, each fecal sample was extracted using a mixture solution of 0.25 M NaOH and 0.05 M EDTA for 16 h with a solid/solution ratio of 1/10, followed by centrifugation at 11000 rpm for 20 min at 4 °C and immediately lyophilized [27]. A total of 300 mg of the lyophilized extract was redissolved in a mixture of 0.1 mL 10 M NaOH, 0.6 mL D_2_O, and 0.02 mL 0.11 M bicarbonate-buffered dithionite solution [26]. Then transferred into a 5 mm NMR tube for ^31^P NMR spectroscopy measurement using a Bruker 400M spectrometer (Switzerland). The spectra parameters were set as an observation pulse of 45^◦^, an acquisition time of 0.7 s, a relaxation delay of 3.0 s, and scans of 20,000 (collected over 20 h). The chemical shift for each fraction was corrected using an 85% H_3_PO_4_ solution. Individual signal peak areas for each P_o_ compound were identified and integrated [26].

### DNA extraction, metagenomic sequencing and bioinformatics analysis

Genomic DNA was extracted from 0.5 g of each fecal sample using the PowerSoil DNA Isolation Kit (MO BIO, USA) following the manufacturer’s protocol. Shotgun metagenomic sequencing was conducted on the Illumina NovaSeq 6000 platform with a paired-end 150 bp (PE150) strategy at Majorbio BioPharm Technology Co., Ltd. (Shanghai, China). Each sample achieved a minimum sequencing depth of 6 Gb. Raw reads were processed for quality control using fastp (v0.20.0), including adapter trimming, low-quality base removal (Q < 20), and read-length filtering (minimum length ≥ 50 bp) [28]. Metagenomes were then de novo assembled from quality-controlled reads using MEGAHIT (v 1.1.2). Contigs of at least 300 bp in length were retained for open reading frame (ORF) prediction using Prodigal (v 2.6.3). Gene clustering was performed using CD-HIT (v4.6.1), with the longest sequence in each cluster selected as the representative to construct a non-redundant gene catalog. Taxonomic classification, pathogens identification, and functional annotation of ARGs, MGEs and VFs were conducted by aligning the gene catalog against the NR database (v 202209), PHI-base, CARD (v3.0.9), MGEs90, VFDB (v20200703) [22]. Alignment thresholds were set at an e-value ≤ 10^−5^. To mitigate interference from dietary  .plant and animal residues, sequences assigned to the kingdoms Viridiplantae and Metazoa—as well as those classified only at higher taxonomic ranks (e.g., “unclassified” or “uncultured”)—were rigorously excluded during species-level annotation [29]. Raw reads of DNA sequence files were uploaded to the China National Center for Bioinformation (CNCB) database under BioProject PRJCA065986, and the accession number of CRA044224.

### Statistical analysis

Data statistics and Spearman correlation analysis were conducted using Excel 2016. Geographic mapping was done using ArcGIS 10.2, and data visualization was plotted using Origin 2024b software. Co-occurrence networks were constructed using the ten most abundant taxa, selected independently for ARGs, VFs, MGEs, and pathogens, based on their relative abundance. Pairwise correlations were computed using the Hmisc package in R[25]. Only Spearman correlations with |r| ≥ 0.75 and significance *P* < 0.05 were retained for co-occurrence network construction; the resulting networks were visualized in Gephi (v0.9.2). Unless otherwise specified, default parameters were used for the statistical packages.

## Results

### Physicochemical Properties of Bird Feces

The physicochemical properties of MBF are summarized in S1 Table. The mean elemental concentrations (dry weight) were as follows: carbon (C) 32.36 ± 5.67%; nitrogen (N) 3.09 ± 0.75%; hydrogen (H) 4.57 ± 0.67%; calcium (Ca) 4.15 ± 1.32%; potassium (K) 1.56 ± 0.47%; aluminum (Al) 1.47 ± 1.33%; and iron (Fe) 1.29 ± 0.91%. Nutrient element P concentrations were obviously higher in the feces of Gruiformes species (*G. nigricollis*, *G. grus,* and *F. atra*) with 0.65 ± 0.33% (dry weight), than those of Anseriformes species (*A. strepera, A. zonorhyncha, M. penelope,* and *T. ferruginea*) with 0.36 ± 0.07% (dry weight). Numerous metals were detected in bird feces, with average concentrations of zinc (Zn) at 89.81 ± 37.47 mg/kg, nickel (Ni) at 45.13 ± 17.45 mg/kg, chromium (Cr) at 26.38 ± 14.32 mg/kg, copper (Cu) at 10.95 ± 8.99 mg/kg, cadmium (Cd) at 1.82 ± 0.70 mg/kg, and arsenic (As) at 1.61 ± 1.17 mg/kg. Concentrations of Zn, Ni, Cr, Cu, and As were higher in Gruiformes feces, whereas Cd levels were elevated in Anseriformes feces. The nickel concentration in the feces of migratory birds from Caohai Wetland (45.13 ± 17.45 mg/kg) was obviously higher than that reported for seabirds (8.50 mg/kg) [14], penguins(3.33 mg/kg) [30], and seagulls (2.56 mg/kg) [13]. Furthermore, all measured heavy metal concentrations in the MBF, except for Ni, were below the regional background values for farmland soils in the Caohai region [31].

### Phosphorus speciation from SMT and ivanoff extraction

**Fig 2:**
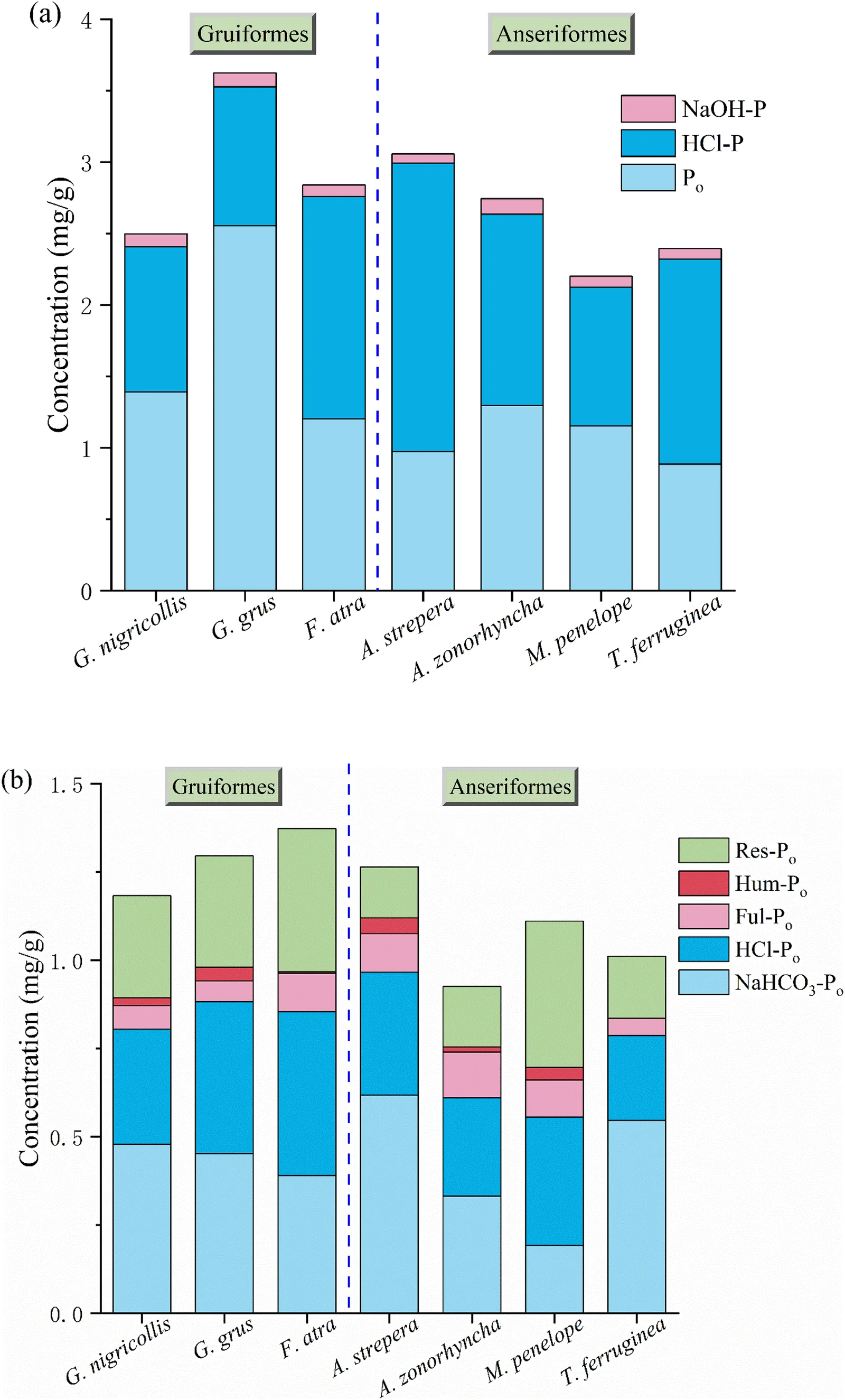
Distribution of phosphorus fractions in MBF determined by SMT extraction procedure (a) and Ivanoff methods (b).

The distribution of various P forms in MBF based on SMT extraction is shown in Fig 2a. The P speciation across all samples followed a consistent pattern: HCl-P > P_o_ > NaOH-P. HCl-P, which is primarily associated with calcium and magnesium compounds, exhibited concentrations ranging from 0.97 to 2.02 mg/g and is generally less bioavailable to plants. Although anaerobic digestion of bird manure provides clear environmental advantages by reducing phosphorus loss, it may concurrently enrich the digestate in metal-bound P forms. HCl-P concentrations were higher in Anseriformes (1.44 mg/g) than in Gruiformes (1.18 mg/g). The P_o_ in feces primarily comprises complex components such as phospholipids, DNA, and phytate [32]. P_o_ concentrations ranged from 0.89 to 2.55 mg/g, with higher levels observed in Gruiformes (1.72 mg/g) than in Anseriformes (1.08 mg/g). NaOH–P (P bound to Al, Fe, and Mn oxides and hydroxides) is considered a potentially bioavailable P fraction. Under anaerobic conditions, this P fraction can be released and may contribute to algal blooms [33]. NaOH–P concentrations ranged from 0.06 to 0.11 mg/g, and no significant difference was found between Gruiformes and Anseriformes feces.

Fig 2b illustrates the distribution of P_o_ fractions in bird feces. The total organic phosphorus content, defined as the sum of all P_o_ forms, was higher in Gruiformes (1.28 mg/g) than in Anseriformes (1.08 mg/g). In terms of the P_o_ form composition, the labile fraction (NaHCO_3_-P_o_) was predominant, followed by the moderately labile fractions (Ful-P_o_ and HCl-P_o_), whereas the non-labile P_o_ fractions (Hum-P_o_ and Res-P_o_) were present in the lowest proportions. NaHCO_3_-P_o_ primarily includes nucleic acids, phospholipids, and phosphosaccharides, and it is highly labile and easily affected by mineralization and decomposition [27]. This indicated that labile and moderately labile P_o_ fractions might be released into the aquatic environment during avian digestive processes.

### Phosphorus character based on ^31^P-NMR

^31^P NMR analysis (S1 Fig) of the bird feces identified four primary classes of P compounds: orthophosphate (Ortho-P, 6.0–7.0 ppm), phosphate monoesters (Mono-P, 4.0–6.0 ppm), DNA-P (−1 ppm), and pyrophosphate (Pyro-P, −4.5 ppm) [34]. The concentrations of these P forms in the MBF, from highest to lowest, were Ortho-P > Mono-P> Pyro-P > DNA-P.

**Fig 3:**
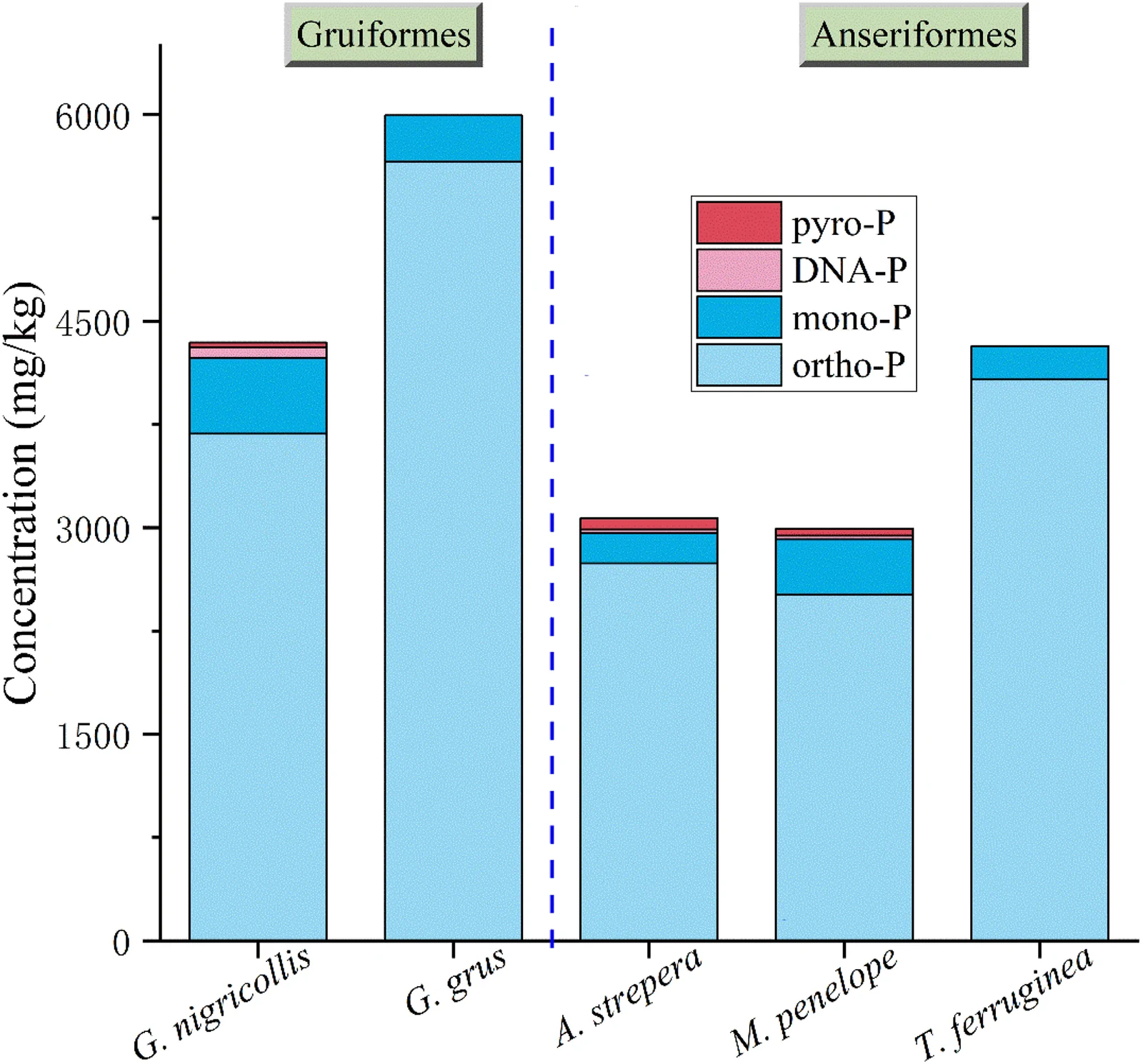
Concentrations of P species by solution ^31^P NMR spectroscopy.

Ortho-P was the dominant P species in feces with an average proportion of 90.68% of total phosphorus (TP), with concentrations ranging from 2514.52 to 5655.33 mg/kg (Fig 3). As the most bioavailable inorganic P form, Ortho-P is readily released from fecal material into overlying water, thereby representing a major contributor to phosphorus loading and subsequent lake eutrophication [10]. This high Ortho-P abundance underscores the substantial risk of rapid P mobilization into the aquatic environment [35]. The concentration of Pyro-P ranged from 0.00 to 82.20 mg/kg, accounting for 0–2.68% of the TP. The relatively low concentration and proportion of Pyro-P in feces suggested partial hydrolysis into orthophosphate, which might support phytoplankton growth. Pyro-P, an inorganic short-chain polyphosphate derived from P esters or hydrolyzed polyphosphates, is a highly unstable compound that can be hydrolyzed into orthophosphate under appropriate conditions, thereby serving as a bioavailable phosphorus source [33]. Moreover, polyphosphate was not detected in feces, likely due to its decomposition into Pyro-P [11]. Despite its low concentration, Pyro-P's high chemical reactivity significantly influenced P dynamics in the environment.

Mono-P constituted the primary form of OP, with concentrations of 219.20–552.20 mg/kg. As shown in the NMR spectra (S1 Fig), the Mono-P region (3.7–4.5 ppm) exhibited 3–4 distinct peaks, which might represent β-glycerophosphate and myoinositol hexakisphosphate (IP_6_) [36]. This observation could potentially be attributed to the degradation of less stable phosphorus-containing compounds, such as RNA-P and phospholipids [26,27]. Diester P, primarily derived from microbial biomass and humic substances, is typically composed of RNA-P, DNA-P, and phospholipids [37]. Due to their lower charge density and greater mobility compared to mono-P, diester P compounds are readily broken down into bioavailable forms [33]. RNA-P and phospholipids were below detection limits across the samples, likely due to their decomposition under intestinal conditions of birds or during the NMR analysis process. The DNA-P content in the feces ranged from 0 to 73.63 mg/kg, with the proportion of 0 to 1.69%. DNA-P is closely associated with microbial biomass and can be a potential indicator of microbial abundance [27]. The detectable DNA-P levels indicate a substantial active microbial community in bird feces, which potentially played a crucial role in P cycling.

### Microbial Community Characteristics

**Fig 4:**
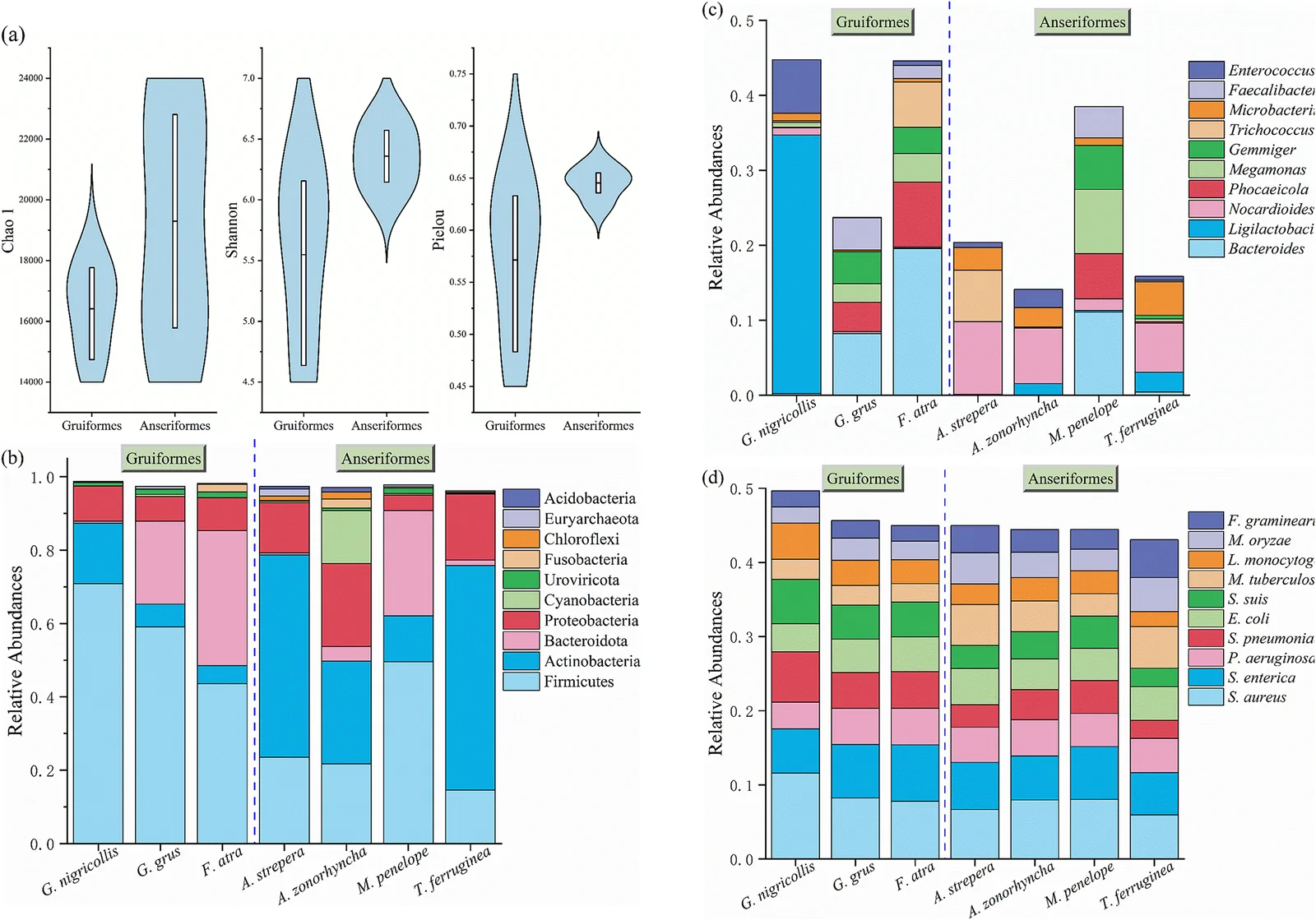
Microbial community characteristics of bird feces.

The detailed sequencing information were provided in S2 Table. Alpha diversity analyses (Chao1, Shannon-Wiener, and Pielou’s evenness) revealed that Anseriformes feces exhibited higher microbial diversity compared to Gruiformes feces (Fig 4a). Taxonomic assignment of high-quality sequencing reads was mapped to 5 domains, 207 phyla, 391 classes, 755 orders, 1568 families, 5472 genera, and 34698 species. Among these microbiomes, bacteria were the predominant microorganisms, accounting for the proportion of 96.63%−98.72%. At the phylum level, Firmicutes accounted for the largest proportion (14.53%−70.86%), followed by Actinobacteria, Bacteroidota, Proteobacteria, and Cyanobacteria (Fig 4b). At the genus level, *Bacteroides*, *Ligilactobacillus, Nocardioides, Phocaeicola, and Megamonas* were the dominant genera (Fig 4c).

A total of 231 potential pathogens were identified, with *Staphylococcus aureus, Salmonella enterica, Pseudomonas aeruginosa, Streptococcus pneumoniae, Escherichia coli, and Streptococcus suis* as the predominant species (relative abundance >4.0%) (Fig 4d). The above-mentioned potential pathogens were recognized as human pathogens. Migratory birds tend to harbor a more diverse total pathogen assemblage than resident birds but do not necessarily carry a higher proportion of zoonoses, implying that migration enhances pathogen acquisition and community mixing, rather than selectively amplifying zoonotic risk [8]. It remains unclear whether these risk-associated bacteria harbor intrinsic resistance phenotypes or have acquired resistance mechanisms through horizontal gene transfer or other evolutionary processes. Given these findings, we recommend implementing sustained, long-term surveillance of drug-resistant bacteria in the region to inform public health strategies and mitigate potential outbreaks.

### ARGs, MGEs, and VFs in bird feces

**Fig 5:**
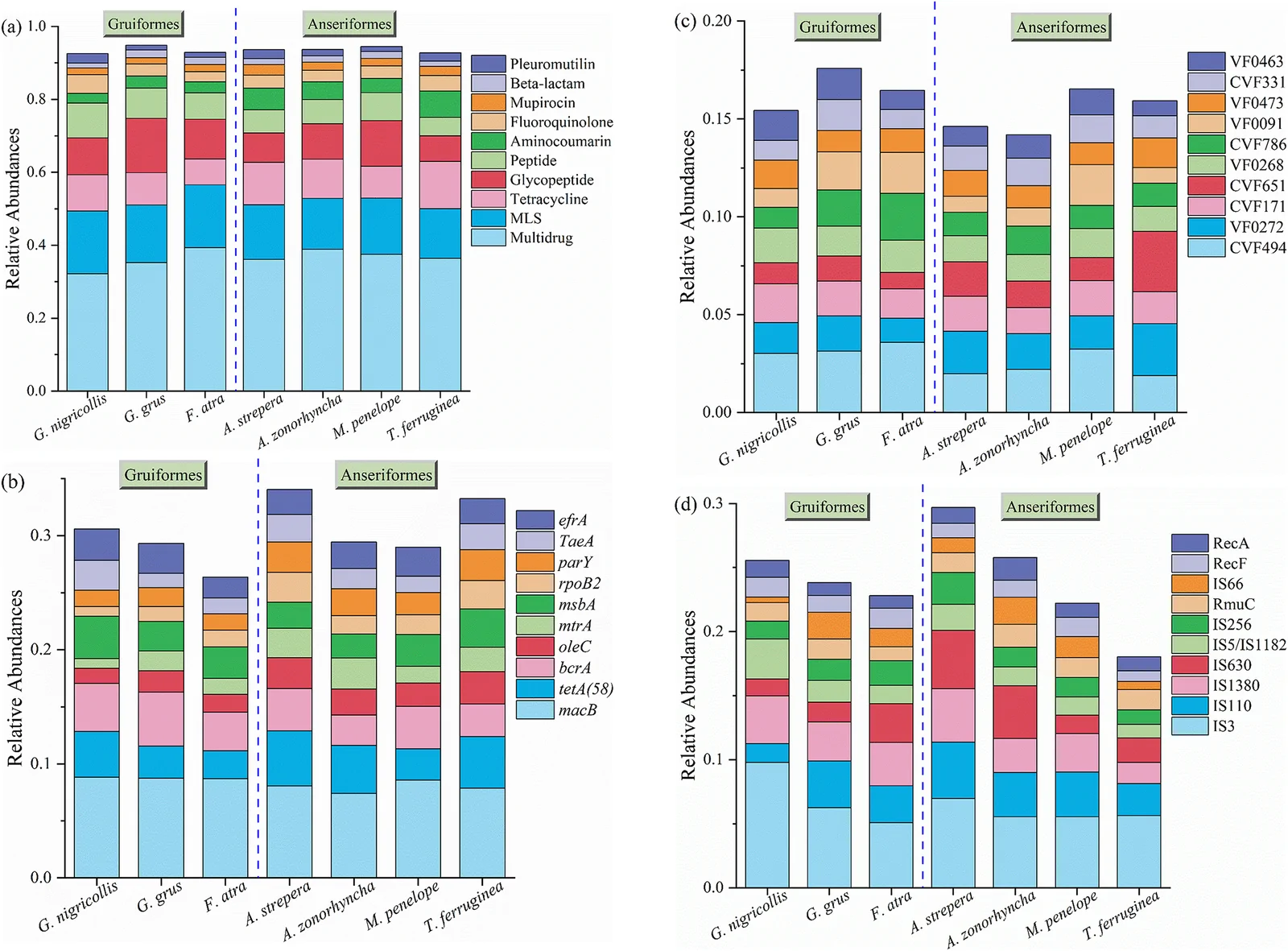
The composition of risk factors in bird feces.

The composition of ARGs at the resistance class and gene subtype levels is presented in Fig 5a and 5b. Among the 21 identified resistance classes, multidrug (32.14–39.29%), macrolide–lincosamide–streptogramin (MLS, 14.01–17.28%), glycopeptide (7.03–14.92%), and tetracycline (7.03–12.95%) constituted the predominant ARG components. The macB gene was the most prevalent ARG subtype in feces and maintained consistently high abundance across all birds. *tetA*(58), *bcrA, oleC,* and *novA* also displayed relatively high abundance. *macB* encodes an MLS resistance efflux pump and is ubiquitously distributed across diverse environmental resistomes [28,38]. The clinically critical and last-line antibiotic tigecycline resistance gene *tet(X3)* pose a serious global threat to public health, and have been detected in migratory birds [39].

VFs enable bacteria to colonize host organisms, evade or suppress immune defenses, and initiate disease processes; thus, research on VFs is essential for a comprehensive understanding of bacterial pathogenicity [22]. To complement the characterization of ARGs, a screening of metagenomic assemblies against the Virulence Factor Database (VFDB) was conducted. Functional category analysis of VFs (Fig 5c) demonstrated that the iron uptake system (20.78%), adherence, secretion system, regulation, antiphagocytosis, and toxin were the predominant categories shared across all samples, with each exhibiting abundance above 10%. Specific VFs exhibited higher dominance in bird feces samples, including LOS (CVF494), FbpABC (VF0272), and beta-hemolysin/cytolysin (CVF171).

MGEs were the key factors influencing ARGs’ diversity and distribution [16]. Transposase was the predominant type of MGEs, accounting for 67.04% across all fecal samples, followed by recombinase, integrase, and conjugative transfer protein, each exhibiting a relative abundance higher than 1% (Fig 5d). Specifically, IS3, IS110, IS1380, IS630, IS5/IS1182, IS256, RmuC, IS66, RecF, and RecA showed strong enrichment in MBF. As the most abundant class of MGEs in bacterial genomes, IS elements facilitate horizontal transfer by inserting into diverse genomic contexts, capturing and mobilizing ARG/VF cassettes, and mediating recombination between plasmids and chromosomes [40,41].

### Correlation analysis

**Fig 6:**
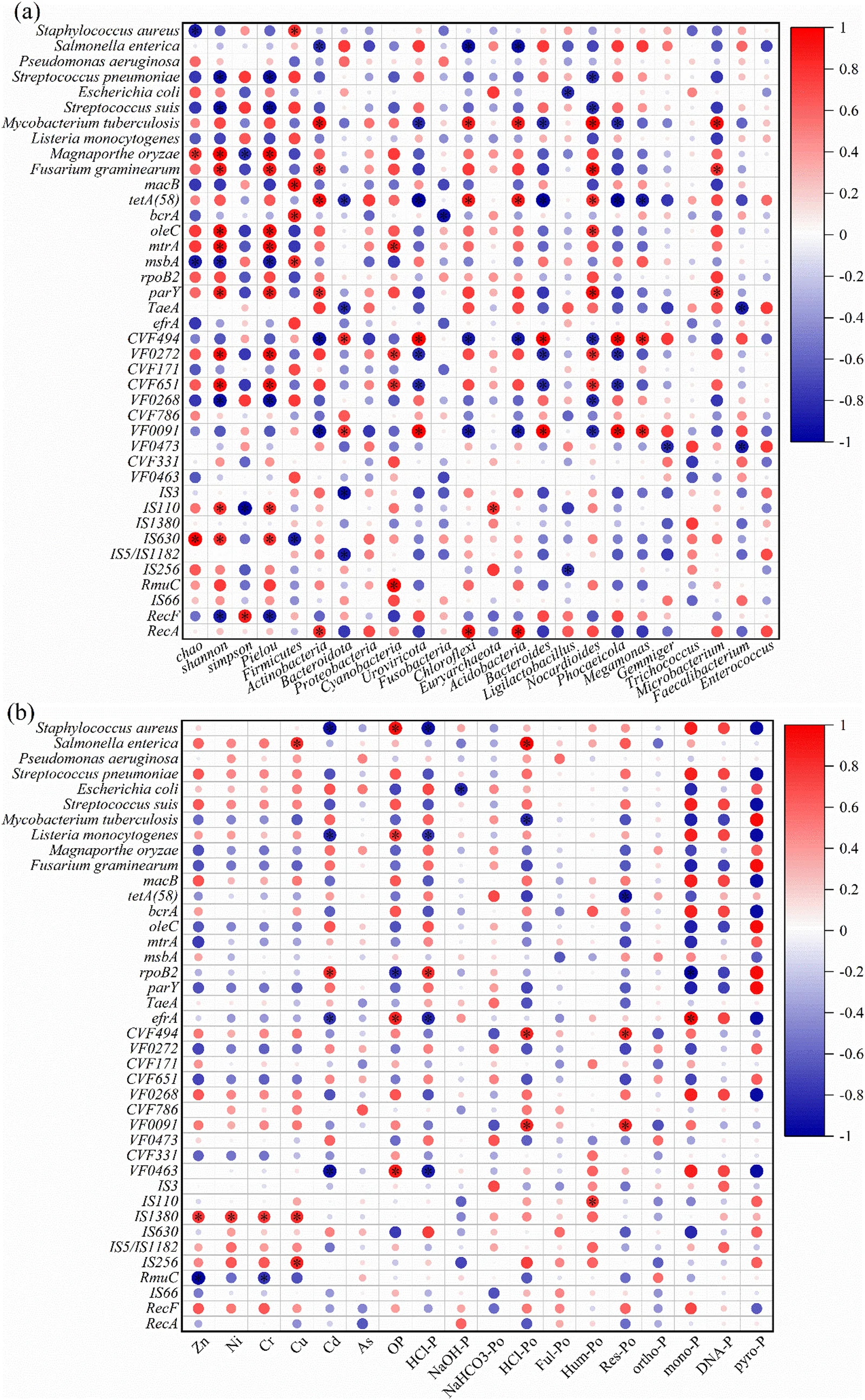
Heatmap showing correlations among (a) microbial community composition and ecological risk factors, and (b) phosphorus fractions and environmental factors with ecological risk factors.

**Fig 7:**
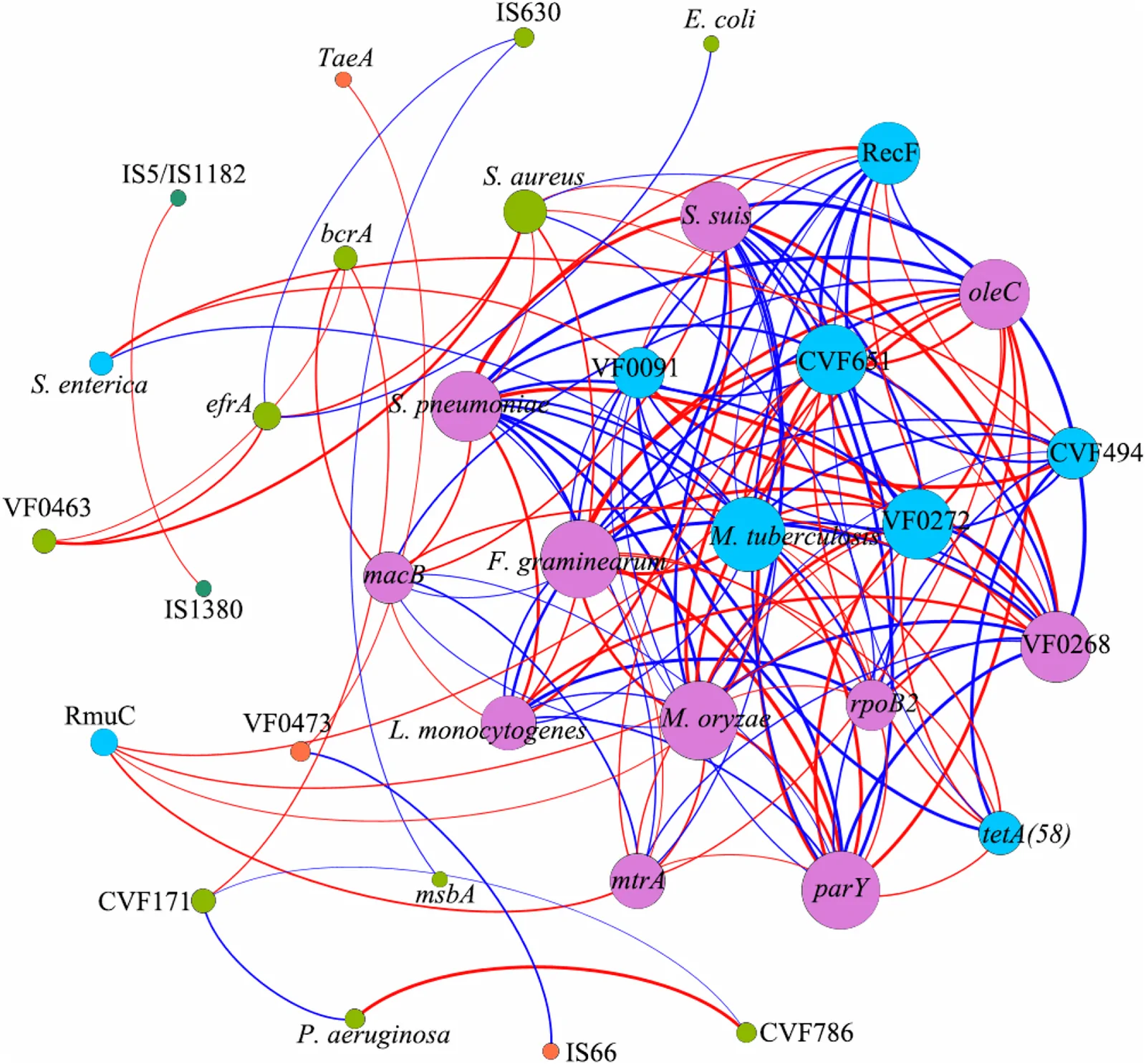
Co-occurrence network analysis of ARGs, VFs, MGEs, and pathogens in feces.

As shown in Fig 6a, the microbial community exhibited an inverse correlation with the abundance of pathogenic bacteria, generally. Intriguingly, a positive correlation between plant pathogenic fungi (*Magnaporthe oryzae*, *Fusarium graminearum*) and alpha diversity indices (Shannon, Pielou evenness) was observed in bird feces, suggesting potential ecological adaptability of these fungi within complex gut microbial communities. Firmicutes, a dominant phylum in avian gut microbiota, exhibited significant positive correlations with ARGs (*macB, bcrA, msbA*), which aligns with its role as a key reservoir of ARGs in diverse microbiomes. Actinobacteria, particularly the genus *Nocardioides*, emerged as a key vector for ARGs (*tetA*(58), *parY*). Additionally, significant correlations were observed between specific microbial taxa (Bacteroidota, Acidobacteria, Uroviricota, and *Nocardioides, Microbacterium*) and key VFs, including CVF494, VF0272, CVF651, and VF0091. MGEs such as IS110, IS630, and *RecF* exhibited strong positive correlations with both microbial alpha diversity and ARGs abundance, indicating their pervasive role in structuring resistome composition. Significant correlations (Fig 6b) were observed between Cu/Cd concentrations and key functional genetic elements: the RNA polymerase subunit gene *rpoB2* associated with rifamycin resistance [22], the multidrug efflux pump gene *efrA*, the virulence factor VF0463 (involved in adhesion and invasion), and the mobile genetic elements IS1380 and IS256. Stable P pools, including HCl-P_o_ and Res-P_o_ showed significant correlation with VF0091, CVF494, *texA*(58). While highly labile P forms, monophosphate (Mono-P), nucleic acid phosphorus (DNA-P), and pyrophosphate (Pyro-P), exhibited strong positive correlations (|r| > 0.5) with both high-risk pathogens (*Escherichia coli, Streptococcus pneumoniae, Listeria monocytogenes*) and high-connectivity ARGs (*macB, bcrA, oleC*). The co-occurrence network structure integrating ARGs, VFs, MGEs, and pathogenic bacteria is displayed in Fig 7. This network included 35 nodes and 141 edges, with positive interactions of 51.06%. Key topological metrics include an average degree of 8.06, an average clustering coefficient of 0.675, and an average path length of 2.52. *ParY, Fusarium graminearum,* and Magnaporthe oryzae emerged as the top-degree hub nodes (degree = 17) in the network.

## Discussion

### Microbial community linked to ecological risk factors

The gut microbiota of migratory birds functions as a critical interface linking host health to ecological risk dissemination, with its taxonomic composition and functional potential directly shaping the expression of ARGs, VFs, and pathogenic taxa. Consistent with the “microbial homeostasis” phenomenon [24], numerous microbial communities exhibited inverse correlations with the abundance of pathogenic bacteria (Fig 6a). The commensal taxa limit the niche availability of opportunistic pathogens through mechanisms including nutrient competition and the production of antimicrobial metabolites (e.g., Actinobacteria) [42]. Firmicutes serve as a key reservoir of ARGs in diverse microbiomes. This association might be driven by their capacity for endospore formation, which enhances bacterial persistence under environmental stress [9], and the prevalence of conjugative plasmids and transposons that facilitate vertical and horizontal ARG transfer. Ecologically, Firmicutes contribute to host energy acquisition and nutrient metabolism through the breakdown of carbohydrates, polysaccharides, and fatty acids, while their abundance is positively linked to immune function [21]. Additionally, Firmicutes possess metabolic pathways associated with extra-host survival mechanisms, cell adhesion, colonization, and host transmission, such as sporulation, glycan biosynthesis, bile acid metabolism, and short-chain fatty acid production, underscoring their functional capabilities and adaptive strategies in vertebrate hosts [9]. Actinobacteria also emerged as a key vector for ARGs. This observation is consistent with the phylum’s evolutionary adaptation to produce secondary metabolites, such as antibiotics [42]. These associations suggest that these taxa may function as “pathogenicity enhancers”: either by directly expressing VFs or by modulating the gut microenvironment to augment the virulence of co-occurring pathogens [43]. For example, iron acquisition, a function mediated by VFs, accounted for 20.78% of all identified VFs in avian fecal samples (Fig 5c).

The high connectivity (Fig 7) observed with pathogens (e.g., *F. graminearum, M. oryzae, S. suis, S. pneumoniae, M. tuberculosis*) and both ARGs and VFs further demonstrated that these taxa frequently co-harbor resistance and virulence determinants [8]. Upon deposition into the wetland, such pathogens pose a high threat of food-web-mediated transmission. This high connectivity underscores that ARGs function not merely as isolated resistance markers but as structural and functional keystones within the ecological risk network. An increasing body of evidence confirms a statistically significant positive association between the distribution of ARGs and key environmental drivers, including bacterial community composition, heavy metal concentrations, antibiotic residues, MGEs, and physicochemical properties [44]. HGT through MGEs is often regarded as a predominant driver in the dissemination and dynamics of ARGs [45]. MGEs not only function as primary vectors for ARG spread but also profoundly influence bacterial pathogenicity. MGEs enhance bacterial metabolic versatility—by horizontally acquiring metabolism-related genes or by modulating the metabolic pathways of host bacteria—thereby expanding substrate utilization breadth and improving metabolic plasticity [46], which collectively strengthen bacterial colonization fitness within the avian intestinal microenvironment. Insertion sequence elements (e.g., IS110, IS630) are small, simple transposons that encode a transposase enabling their self-mobilization, thereby facilitating the horizontal transfer of ARGs and VFs [41]. MGE-mediated effects can include changes in antimicrobial resistance (such as by encoding enzymes that inactivate antibiotics), virulence (such as by encoding toxins), phage susceptibility, defence against other MGEs, sporulation, extracellular polysaccharide (EPS) production and biofilm formation properties [46]. The significant positive correlations between VFs and MGE-associated genes (e.g., RmuC, RecF) provide direct empirical evidence that virulence traits can be horizontally transferred, potentially transforming commensal or environmental bacteria into emergent pathogens and thereby expanding the reservoir of ecologically hazardous strains. Under selective pressures, including nutrient fluctuations and heavy metal exposure, ARGs and VFs are co-mobilized via MGEs across diverse bacterial hosts, synergistically enhancing pathogen fitness, environmental persistence, and transmission potential. Collectively, these results established the avian gut microbiota as a dynamic, functionally integrated hub for the accumulation and environmental dissemination of multifaceted biological risks, including ARGs, VFs, and phytopathogenic taxa. Consequently, migratory bird feces–impacted ecosystems experience systemic amplification of coupled ecological risks: accelerated dissemination of antimicrobial resistance and concurrent enhancement of bacterial virulence.

### Environmental regulation

Heavy metals such as Cu and Cd act as potent abiotic selectors, driving microbial community succession toward taxa that exhibit both metal tolerance and enrichment of ARGs and virulence determinants [47]. This selective pressure is robustly supported by these associations indicated that heavy metals induce cellular stress responses, which concurrently upregulate resistance mechanisms and promote MGE-mediated horizontal gene transfer [18]. The strong positive correlation between heavy metals and the genus *Trichococcus* underscores its intrinsic tolerance to metal stress and positions it as a potential reservoir for ARG persistence and dissemination within the fecal microbiome. This finding aligns with Yu et al [48], who reported that *Trichococcus* exhibited enhanced tolerance to Cd and served as a host for key ARGs, including *tetA, czcA*, and intI1. P speciation functions as a nutrient driver, with its regulatory influence governed by P bioavailability. Stable P pools, including HCl-P_o_ and Res-P_o_, act as slow-release nutrient reservoirs [11]. This sustained nutrient supply promotes the growth of K-strategist bacteria, which are adept at utilizing recalcitrant carbon sources [49]. Gradual mineralization of these stable P fractions supports the growth of ARG-carrying bacteria, thereby promoting enrichment of specific ARGs, including *tetA*(58). Concurrently, the mineralization process yields energy substrates that induce expression of VFs (e.g., VF0091, CVF494), enhancing both the environmental fitness and host infectivity of pathogenic strains. Multidrug efflux pumps and other efflux transporters, such as *macB* play key roles in cell detoxification, bacterial homeostasis, and cell-in-cell communication, may be beneficial for bacteria to efficiently utilize nutrients under N or P limitation conditions [45]. The rapid nutrient influx favors the proliferation of r-strategist bacteria, thereby reshaping the bacterial community structure and function [49]. Such nutrient-mediated shifts in bacterial survival strategies further modulate vertical and horizontal gene transfer of ARGs by regulating host bacterial growth and interspecific bacterial interactions, ultimately reshaping the distribution of ARGs [49]. In contrast, Cao et al [50] reported no significant correlation between microbial community composition and resistome structure in migratory birds, highlighting context-dependent variability in ARG–microbiome associations. Further in-depth investigation is warranted to reconcile these divergent patterns across ecosystems and host taxa. Collectively, these findings demonstrate that heavy metals and P speciation interact synergistically: metal stress selects for resilient, genetically equipped microbes, while P availability fuels their proliferation and functional expression, ultimately steering the fecal microbiome toward a “resistant–pathogenic” phenotype characterized by elevated ARG–VFs co-occurrence.

### Ecological risk assessment and recommendations

Migratory birds are increasingly recognized as significant vectors of pathogen dispersal globally, facilitating the transmission of viral, bacterial, and parasitic agents [8]. Wang et al [23] reported 1814 known ARGs and 7219 VFs in migratory birds. The comprehensive analysis of fecal samples from overwintering migratory birds in Caohai Wetland revealed ecological and public health risks, driven by the synergistic effects of nutrient accumulation, heavy metals, ARGs, VFs, and potential pathogens. Nutritionally, the differential enrichment of N (Anseriformes) and P (Gruiformes) in feces, coupled with the dominance of highly bioavailable Ortho-P and labile organic phosphorus (NaHCO_3_-P_o_), poses a significant eutrophication risk to the overlying water column [36]. The stable phosphorus pool further sustains the proliferation of drug-resistant bacteria and pathogens by providing a continuous nutrient supply. P and N concentrations in migratory bird feces averaged 0.48% and 3.09% on a dry weight basis, respectively (S1 Table). These values are lower than those reported for marine seabird and polar avian guano, where total P reaches up to 15.08 ± 1.07% and total N up to 15.08 ± 1.07% of dry weight [14]. Nevertheless, orthophosphate, the most bioavailable P fraction, accounted for 90.68% of total P and labile NaHCO_3_-P_o_ constituted the dominant organic P fraction, collectively implying that guanotrophication potential cannot be ignored in the Caohai Wetland. Resistome profiling revealed 21 ARG classes, with multidrug, MLS, and glycopeptide resistance genes being more abundant, whereas the nationwide survey [23] identified more clinically critical carbapenem and colistin resistance determinants, absent from Caohai plateau wetland samples. Wang et al [23] suggested that zoonotic pathogens or opportunistic pathogens may spread along with the cross-national and intercontinental migrations of wild birds, posing potential risks to global public health. A total of 231 potential pathogens were identified in MBF. Fang et al [22] reported that *Pseudomonas aeruginosa* and *Salmonella enterica* were the primary pathogens detected in reindeer feces. Li et al [5] identified *Fusarium* as the second most abundant fungal genus in black-necked cranes’ feces. The presence of these potential pathogens may reflect either their capacity to metabolize gut microbiota-derived byproducts or host immunomodulatory mechanisms that tolerate non-pathogenic colonization. Notably, as highly destructive pathogens with broad host ranges, they pose substantial threats to regional biosecurity through avian-mediated long-distance dispersal, which bypasses geographical barriers and enables cross-ecosystem transmission [8,24]. This dispersal pathway also amplifies the risk of zoonotic transmission [21]. Pathogen profiling further demonstrated that zoonotic enteric bacteria—including *Salmonella* spp. and *Escherichia coli*—are consistently present across all wild waterbird microbiome datasets. In contrast, phytopathogenic fungi such as *Fusarium graminearum* and *Magnaporthe oryzae*—detected here for the first time in a waterbird fecal resistome study—have rarely been reported in prior migratory bird microbiome research [5]. Their presence reflects a distinct ecological exposure pathway linked to farmland foraging behavior and represents a previously underrecognized biosafety risk specific to plateau-agricultural ecotones.

Heavy metal contamination, characterized by high concentrations of Zn (81.25 mg/kg), Ni (41.01 mg/kg), and Cr (23.49 mg/kg) in Gruiformes feces and elevated Cd (1.77 mg/kg) and As (1.71 mg/kg) in Anseriformes feces, exerts strong selective pressure that drives the succession of microbial communities toward drug-resistant and pathogenic taxa. This is corroborated by the significant correlations between heavy metals and ARGs (e.g., *efrA*), VFs (e.g., VF0463), and MGEs (e.g., IS1380, IS256). The co-occurrence of predominant ARG classes (multidrug, MLS, glycopeptide, tetracycline) with highly enriched subtypes (*macB, tetA*(58)*, bcrA*), key VFs (iron uptake system, adherence, toxin-related CVF494, VF0272), and MGEs (transposase accounting for 67.04%) facilitates the horizontal transfer and enrichment of resistance and virulence traits. The high connectivity observed in the co-occurrence network of ARGs, VFs, MGEs, and pathogens indicates that these taxa may harbor acquired resistance via MGEs or intrinsic resistance mechanisms. Collectively, a risk cascade linking geochemical and microbial hazards from MBF was identified. Firstly, heavy metal exposure in the avian gut imposes toxicity stress on microbial communities, selecting for hosts carrying both metal tolerance genes and ARGs. Then, induces transposases, integrases, and recombinases, boosting abundance and mobility of insertion sequence MGEs; These mobilizable MGEs capture and co-transfer ARG cassettes and virulence operons. Finally, Co-transfer of resistance and virulence traits confers dual fitness advantages, enabling opportunistic pathogens to outcompete commensals and enrich multi-risk pathogenic populations in fecal deposits. Once deposited in plateau wetlands, labile phosphorus fuels growth and persistence of these resistant-pathogenic assemblages; cold temperatures [27] slow microbial degradation, prolonging the viability of transmissible ARGs and pathogens in water and sediment [8]. These interlinked factors constitute a “nutrient–heavy metal–antibiotic resistance–pathogenicity” synergistic risk network, threatening both aquatic ecosystem stability and public health.

This study highlights the critical role of migratory birds in the transmission of emerging infectious diseases, consistent with the conclusions of previous related studies [14,51]. However, four key limitations warrant consideration: (1) Lack of paired environmental matrices: Only fecal samples were analyzed; concurrent water and sediment samples were not collected, preventing quantification of bird-derived phosphorus, ARGs, and pathogens in wetland habitats. (2) Spatially restricted sampling: All samples originated from the Caohai Wetland, limiting generalizability to other plateau wetlands. (3) Limited metagenomic sequencing depth and replication: both the number of biological replicates per bird species and sequencing depth were insufficient to robustly resolve low-abundance ARGs and pathogen communities. (4)Absence of in situ validation: no field microcosm or mesocosm experiments assessed the actual transmission efficiency of ARGs and pathogens from bird feces to resident wetland microbial communities.

From an integrated One Health perspective, migratory waterbirds act as interconnected biological vectors linking wildlife ecosystems, agricultural production zones, and human residential environments [4]. Migratory birds assemblages fulfill a dual ecological role: they play a pivotal role in seed dispersal, pest control, and nutrient cycling, thereby contributing to increased agricultural productivity and enhanced ecosystem resilience [8]; they also act as reservoirs of multifaceted biological hazards. This duality necessitates balanced, multi-stakeholder management frameworks. In response to wild bird conservation needs, China has implemented comprehensive strategies targeting both population viability and habitat integrity [24]. Besides, Targeted wetland management recommendations were proposed. (1) Prioritize habitat protection and ecological restoration, specifically by conserving native plant diversity, improving waterbird habitat quality, and mitigating anthropogenic disturbance and invasive species impacts [52]. (2) Establish a long-term surveillance system to monitor spatiotemporal dynamics of ARGs and pathogens in the gut microbiota and surrounding wetland environment [24]. (3) Promote interdisciplinary collaboration by formalizing sustained coordination among ecologists, microbiologists, environmental toxicologists, and public health specialists. Facilitate interdisciplinary collaboration between wetland ecologists, wildlife conservationists, environmental microbiologists, and local public health authorities to develop plateau wetland biosecurity protocols. This coordination of conservation strategies and risk mitigation measures was rigorously grounded in the integrated One Health framework [8,18].

## Conclusions

1. Migratory bird feces are important sources of nutrients in plateau wetlands. P speciation is dominated by highly bioavailable ortho-P and labile organic phosphorus (NaHCO_3_-P_o_), posing guanotrophication risk for shallow plateau lake ecosystems.
2. Bird feces harbored diverse and abundant ARGs, VFs, and potential pathogens. A total of 231 potential pathogens, including *S. aureus, S. enterica, P. aeruginosa* were identified. Multidrug, MLS, glycopeptide, and tetracycline resistance genes were the major ARG classes, and the subtypes macB, tetA(58), and bcrA were highly enriched.
3. Heavy metals (abiotic selectors) and P speciation (nutrient driver) act synergistically to shape fecal microbiome succession toward multi-resistant pathogenic taxa; concurrently, low plateau temperatures further prolong the environmental persistence of resistance and pathogenic contaminants excreted via bird feces.
4. This study reveals a plateau-specific “nutrient–heavy metal–resistance–pathogenicity” synergistic risk network driven by migratory bird fecal inputs. Integrated management recommendations grounded in the One Health framework were proposed.

## Supporting information

S1 Table The physicochemical properties of MBF. (DOCX)

S2 Table The detailed sequencing information of MBF. (DOCX)

S1 Fig The spectra by ^31^P NMR spectroscopy of bird feces. (TIF)

## References

[1] Dai S, Zhou Y, Gao J, Cheng G, Wang L, Bai H, et al. The preference of migratory waterbirds to winter at a key location on the East Asian-Australasian flyway and its implications for habitat conservation. Ecol Inform. 2025;85:102935. doi: 10.1016/j.ecoinf.2024.102935

[2] Hu W, Zhang Z, Mu G. Revealing the ecological transitions and driving mechanisms in plateau lake wetlands conservation through a three-decade landscape ecology analysis. J Clean Product. 2025;495:145066. doi: 10.1016/j.jclepro.2025.145066

[3] Zhong L, Li Y, Li Y, Dai C. Hungry wintering birds and angry farmers: Crop damage and management implications in a protected wetland in China. Glob Ecol Conserv. 2025;57:e03402. doi: 10.1016/j.gecco.2025.e03402

[4] Adhurya S, Ray S. Scenario analysis with a simulation model of the effect of waterbirds on phosphorus dynamics of a shallow freshwater ecosystem. Environ Chall. 2022;9:100618. doi: 10.1016/j.envc.2022.100618

[5] Li W, Cheng L, He X, He G, Liu Y, Sang Z, et al. Gut fungi of black-necked cranes (Grus nigricollis) respond to dietary changes during wintering. BMC Microbiol. 2024;24(1):232. doi: 10.1186/s12866-024-03396-0 38951807 PMC11218170

[6] Otero XL, De La Peña-Lastra S, Pérez-Alberti A, Ferreira TO, Huerta-Diaz MA. Seabird colonies as important global drivers in the nitrogen and phosphorus cycles. Nat Commun. 2018;9(1):246. doi: 10.1038/s41467-017-02446-8 29362437 PMC5780392

[7] Boros E, Takacs A, Dobosy P, Voros L. Extreme guanotrophication by phosphorus in contradiction with the productivity of alkaline soda pan ecosystems. Sci Total Environ. 2021;793:148300. doi: 10.1016/j.scitotenv.2021.148300 34174614

[8] Olowu BI, Zakariya ME, Azeez AO, Al-Awal AA, Adebayo KS, Idris NO, et al. Propagation of Emerging and Re-Emerging Infectious Disease Pathogens in Africa: The Role of Migratory Birds. Bacteria. 2026;5(1):2. doi: 10.3390/bacteria5010002

[9] Dias B do C, Lamarca AP, Machado DT, Kloh VP, de Carvalho FM, Vasconcelos ATR. Metabolic pathways associated with Firmicutes prevalence in the gut of multiple livestock animals and humans. Anim Microbiome. 2025;7(1):20. doi: 10.1186/s42523-025-00379-y 40033444 PMC11874851

[10] Zheng Z, Wang X, Jin J, Hao J, Nie Y, Chen X, et al. Fraction distribution and dynamic cycling of phosphorus in lacustrine sediment at Inexpressible Island, Antarctica. Environ Int. 2022;164:107228. doi: 10.1016/j.envint.2022.107228 35468407

[11] Long Y, Wang L, Hu J, Jiang J. Biogeochemical characteristics and bioavailability of organic phosphorus in sediments of degrading plateau lake Caohai: Implications for internal phosphorus loading and eutrophication risk. Water Air Soil Pollut. 2026;237(3):161. doi: 10.1007/s11270-025-08883-2

[12] Han H, Liu H, Zhang B, Li Y, Li C, Cao H. Competitive relationships due to similar nutrient preferences reshape soil bacterial metacommunities. Sci Total Environ. 2024;933:172956. doi: 10.1016/j.scitotenv.2024.172956 38719036

[13] Alba-González P, Álvarez-Salgado XA, Cobelo-García A, Kaal J, Teira E. Faeces of marine birds and mammals as substrates for microbial plankton communities. Mar Environ Res. 2022;174:105560. doi: 10.1016/j.marenvres.2022.105560 35021141

[14] Grant ML, Bond AL, Lavers JL. The influence of seabirds on their breeding, roosting and nesting grounds: A systematic review and meta-analysis. J Anim Ecol. 2022;91(6):1266–89. doi: 10.1111/1365-2656.13699 35395097 PMC9324971

[15] Watson E, Hamilton S, Silva N, Moss S, Watkins C, Baily J, et al. Variations in antimicrobial resistance genes present in the rectal faeces of seals in Scottish and Liverpool Bay coastal waters. Environ Pollut. 2024;349:123936. doi: 10.1016/j.envpol.2024.123936 38588972

[16] Zhou Z, Zhu R, Song Y, Zhang W, Sun B, Zhang Z, et al. Penguin-Driven Dissemination and High Enrichment of Antibiotic Resistance Genes in Lake Sediments across Antarctica. Environ Sci Technol. 2024;58(32):14460–74. doi: 10.1021/acs.est.4c02732 39083437

[17] Robins K, O’Donnell G, Neumann A, Schmidt W, Hart A, Graham DW. Antimicrobial resistance in rural rivers: Comparative study of the Coquet (Northumberland) and Eden (Cumbria) River catchments. Sci Total Environ. 2024;928:172348. doi: 10.1016/j.scitotenv.2024.172348 38614353

[18] Wu Z, Shao X, Wang Q. Antibiotics and Antibiotic Resistance Genes in the Environment: Dissemination, Ecological Risks, and Remediation Approaches. Microorganisms. 2025;13(8):1763. doi: 10.3390/microorganisms13081763 40871267 PMC12388852

[19] Jarma D, Sacristán-Soriano O, Borrego CM, Hortas F, Peralta-Sánchez JM, Balcázar JL, et al. Variability of faecal microbiota and antibiotic resistance genes in flocks of migratory gulls and comparison with the surrounding environment. Environ Pollut. 2024;359:124563. doi: 10.1016/j.envpol.2024.124563 39019307

[20] Sacristán-Soriano O, Jarma D, Sánchez MI, Romero N, Alonso E, Green AJ, et al. Winged resistance: Storks and gulls increase carriage of antibiotic resistance by shifting from paddy fields to landfills. Sci Total Environ. 2024;914:169946. doi: 10.1016/j.scitotenv.2024.169946 38199372

[21] Yang F, He S, Xu W, Sun K, Jin L, Wang H. Gut microbiota and antibiotic resistance genes in endangered migratory Scaly-sided merganser (Mergus squamatus) in northeast China. Glob Ecol Conserv. 2024;55:e03233. doi: 10.1016/j.gecco.2024.e03233

[22] Fang X-M, Li J, Wang N-F, Zhang T, Yu L-Y. Metagenomics uncovers microbiome and resistome in soil and reindeer faeces from Ny-Ålesund (Svalbard, High Arctic). Environ Res. 2024;262(Pt 1):119788. doi: 10.1016/j.envres.2024.119788 39159777

[23] Wang Y, Wu H, Qu M, Zhang C, Xu Z, Pei Y, et al. A Genomic Catalog of Migratory Microbiomes from Wild Birds across China’s Habitats. Adv Sci (Weinh). 2026;13(26):e74581. doi: 10.1002/advs.74581 41742841 PMC13159146

[24] Wang R, Wang Y, Deng L, Wang B, Shi M, Yang Z, et al. Comparative analysis of fecal microbiota of central and eastern black-necked cranes (Grus nigricollis) wintering in Yunnan Province, China. PeerJ. 2025;13:e19520. doi: 10.7717/peerj.19520 40452926 PMC12124291

[25] Long Y, Guo J, Dai L, Jiang J. Interactions between phytoplankton and bacterioplankton communities in Caohai plateau lake, revealed by environmental DNA metagenomics. BMC Microbiol. 2026;26(1):4. doi: 10.1186/s12866-025-04536-w 41491676 PMC12772069

[26] Yuan H, Yuan Q, Guan T, Cai Y, Liu E, Ji M, et al. Regime difference trigger the regulation of mineralization progress of organic phosphorus in lake sediments. Water Res. 2025;284:124002. doi: 10.1016/j.watres.2025.124002 40516401

[27] Long Y, Jiang J, Wu B, Hu J, Zhang Z, Zhou S. Phosphatase phoD gene community changes organic phosphorus in sediment from Caohai plateau wetland. J Soils Sediments. 2022;22(8):2317–28. doi: 10.1007/s11368-022-03245-5

[28] Zhong Y, Wu C, Yao Z, Li X, Chi H, Wu T, et al. Metagenomic Analysis of Distribution Characteristics and Driving Mechanisms of Antibiotic Resistance Genes, Virulence Factors, and Microbial Communities in Rice Seedling Cultivation Soils. Microorganisms. 2025;13(11):2419. doi: 10.3390/microorganisms13112419 41304105 PMC12654301

[29] Chen G, Wang H, Chang C, Zhang Q, Wang J, Zhang F, et al. Migration pattern of protistan groups in the runoff from slopes covered with biocrusts under rainfall in a semiarid region. Catena. 2024;243:108151. doi: 10.1016/j.catena.2024.108151

[30] Rodrigues WF, de Oliveira FS, Schaefer CEGR, Leite MGP, Pavinato PS. Phosphatization under birds’ activity: Ornithogenesis at different scales on Antarctic Soilscapes. Geoderma. 2021;391:114950. doi: 10.1016/j.geoderma.2021.114950

[31] Sun R, Yang J, Xia P, Wu S, Lin T, Yi Y. Contamination features and ecological risks of heavy metals in the farmland along shoreline of Caohai plateau wetland, China. Chemosphere. 2020;254:126828. doi: 10.1016/j.chemosphere.2020.126828 32334265

[32] Ran X, Li S, Tejaswi Uppuluri NS, Deng Y, Su Y, Huang G, et al. Phosphorus transformation and bioavailability in livestock manure through aerobic composting and anaerobic digestion. Chem Eng J. 2025;505:159285. doi: 10.1016/j.cej.2025.159285

[33] Ran X, Tejaswi Uppuluri NS, Deng Y, Wang S, Ni Z, Hu J, et al. Phosphorus bioavailability and recycling potential in various organic Waste: Assessment by enzymatic hydrolysis and 31P NMR. Bioresour Technol. 2025;416:131790. doi: 10.1016/j.biortech.2024.131790 39522620

[34] Yuan H, Tai Z, Li Q, Zhang F. Characterization and source identification of organic phosphorus in sediments of a hypereutrophic lake. Environ Pollut. 2020;257:113500. doi: 10.1016/j.envpol.2019.113500 31733975

[35] Suo Y, Li T, von Sperber C, Ge L, Cao C, Zhai Z, et al. Low molecular weight organic acids mobilize soil organic phosphorus for enzymatic hydrolysis in a temperate montane peatland. Biogeochemistry. 2025;168(1). doi: 10.1007/s10533-025-01210-1

[36] Yang P, Yang C, Yin H. Dynamics of phosphorus composition in suspended particulate matter from a turbid eutrophic shallow lake (Lake Chaohu, China): Implications for phosphorus cycling and management. Sci Total Environ. 2020;741:140203. doi: 10.1016/j.scitotenv.2020.140203 32570068

[37] Ni Z, Li Y, Wang S. Cognizing and characterizing the organic phosphorus in lake sediments: Advances and challenges. Water Res. 2022;220:118663. doi: 10.1016/j.watres.2022.118663 35661507

[38] Zhuang M, Achmon Y, Cao Y, Liang X, Chen L, Wang H, et al. Distribution of antibiotic resistance genes in the environment. Environ Pollut. 2021;285:117402. doi: 10.1016/j.envpol.2021.117402 34051569

[39] Cao J, Wang J, Wang Y, Wang L, Bi Y, Zhu B, et al. Tigecycline resistance tet(X3) gene is going wild. Biosafety Health. 2020;2(1):9–11. doi: 10.1016/j.bsheal.2020.01.002

[40] Chen C, Li Y, Wu Z, Ruan Y, Long T, Wang X, et al. Cat and dog feces as reservoirs of diverse novel antibiotic resistance genes. Environ Res. 2024;261:119690. doi: 10.1016/j.envres.2024.119690 39068967

[41] Kirsch JM, Hryckowian AJ, Duerkop BA. A metagenomics pipeline reveals insertion sequence-driven evolution of the microbiota. Cell Host Microbe. 2024;32(5):739-754.e4. doi: 10.1016/j.chom.2024.03.005 38565143 PMC11081829

[42] Long Y, Jiang J, Hu X, Zhou J, Hu J, Zhou S. Actinobacterial community in Shuanghe Cave using culture-dependent and -independent approaches. World J Microbiol Biotechnol. 2019;35(10):153. doi: 10.1007/s11274-019-2713-y 31576426

[43] Jyoti, Dey P. Mechanisms and implications of the gut microbial modulation of intestinal metabolic processes. NPJ Metab Health Dis. 2025;3(1):24. doi: 10.1038/s44324-025-00066-1 40604123 PMC12441142

[44] Luo H, Xie K, Dong P, Zhang Y, Ren T, Sui C, et al. Assessing the Risks of Potential Pathogens and Antibiotic Resistance Genes Among Heterogeneous Habitats in a Temperate Estuary Wetland: a Meta-analysis. Microb Ecol. 2025;87(1):172. doi: 10.1007/s00248-024-02484-y 39820498 PMC11739316

[45] Liu Q, Jia J, Hu H, Li X, Zhao Y, Wu C. Nitrogen and phosphorus limitations promoted bacterial nitrate metabolism and propagation of antibiotic resistome in the phycosphere of Auxenochlorella pyrenoidosa. J Hazard Mater. 2024;468:133786. doi: 10.1016/j.jhazmat.2024.133786 38367442

[46] Lang AS, Buchan A, Burrus V. Interactions and evolutionary relationships among bacterial mobile genetic elements. Nat Rev Microbiol. 2025;23(7):423–38. doi: 10.1038/s41579-025-01157-y 40069292

[47] Chen Z, Lv Y, Long Y, Jiang J, Zhou S, Ma S, et al. Levels, Spatial Distribution, and Sources of Heavy Metals in Karst Caohai Wetlands and Their Impact on Bacterial Community Diversity and Symbiotic Patterns, China. Pol J Environ Stud. 2025;35(3):3083–107. doi: 10.15244/pjoes/203357

[48] Yu M-F, Shu B, Li Z, Liu G, Liu W, Yang Y, et al. Co-selective Pressure of Cadmium and Doxycycline on the Antibiotic and Heavy Metal Resistance Genes in Ditch Wetlands. Front Microbiol. 2022;13:820920. doi: 10.3389/fmicb.2022.820920 35250936 PMC8895241

[49] Wang P, Jin Q, Zhu N, Hu B, Gao Y. Fallow practice mitigates antibiotic resistance genes in soil by shifting host bacterial survival strategies. J Hazard Mater. 2025;495:138991. doi: 10.1016/j.jhazmat.2025.138991 40555016

[50] Cao J, Hu Y, Liu F, Wang Y, Bi Y, Lv N, et al. Metagenomic analysis reveals the microbiome and resistome in migratory birds. Microbiome. 2020;8(1):26. doi: 10.1186/s40168-019-0781-8 32122398 PMC7053137

[51] Zhou P, Wang M, DuBay S, Cao Y, Zhang S, Zhang J, et al. Widespread microplastic and nanoplastic contamination in the intestines of birds: A case study from Chengdu, China. J Hazard Mater. 2025;493:138369. doi: 10.1016/j.jhazmat.2025.138369 40286662

[52] Qiu J, Zhang Y, Ma J. Wetland habitats supporting waterbird diversity: Conservation perspective on biodiversity-ecosystem functioning relationship. J Environ Manage. 2024;357:120663. doi: 10.1016/j.jenvman.2024.120663 38552509

