# Supplementary material for: Ecological risk assessment of migratory bird feces in plateau wetland: Phosphorus speciation, ARGs, and pathogens
Source: PLoS One. 2026 Sep 21;21(9):e0348709. doi: 10.1371/journal.pone.0348709 (PMC13592592; doi:10.1371/journal.pone.0348709)
Supplement: Supplementary file 1 [file pone.0348709.s001.docx]

**Table. S1 The physicochemical properties of MBF.**

| Samples | N (%) | C (%) | H (%) | S (%) | P (%) | Ca (%) | K (%) | Al (%) | Fe (%) | Mg (%) | Zn (mg/kg) | Ni (mg/kg) | Cr (mg/kg) | Cu (mg/kg) | Cd (mg/kg) | As (mg/kg) |
| --- | --- | --- | --- | --- | --- | --- | --- | --- | --- | --- | --- | --- | --- | --- | --- | --- |
| *G. nigricollis* | 1.9 | 30.06 | 4.22 | 0.28 | 0.3 | 3.57 | 1.93 | 0.74 | 1.31 | 0.7 | 128.14 | 48.34 | 30.91 | 8.61 | 1.12 | 0.73 |
| *G. grus* | 4.08 | 38.4 | 5.26 | 0.49 | 0.68 | 3.2 | 1.67 | 0.61 | 0.46 | 0.35 | 64.93 | 45.2 | 13.63 | 13.83 | 0.96 | 3.66 |
| *F. atra* | 3.28 | 23.77 | 3.61 | 0.43 | 0.96 | 4.19 | 1.16 | 3.35 | 2.67 | 0.7 | 134.92 | 70.31 | 49.47 | 23.97 | 2.39 | 1.66 |
| *A. strepera* | 2.37 | 27.29 | 3.97 | 0.42 | 0.31 | 5.68 | 0.89 | 3.41 | 2.41 | 0.66 | 122.41 | 59.81 | 36.61 | 20.84 | 2.42 | 1.09 |
| *A. zonorhyncha* | 3.73 | 33.78 | 4.68 | 0.46 | 0.4 | 6.26 | 1.3 | 0.9 | 1.01 | 0.46 | 47.52 | 45.38 | 17.45 | 1.69 | 1.46 | 0.87 |
| *M. penelope* | 3.18 | 34.08 | 4.82 | 0.64 | 0.3 | 3.05 | 2.27 | 1.02 | 0.83 | 0.36 | 76.67 | 27.15 | 28.28 | 6.81 | 1.67 | 0.51 |
| *T. ferruginea* | 3.08 | 39.17 | 5.43 | 0.34 | 0.43 | 3.06 | 1.71 | 0.24 | 0.35 | 0.44 | 54.05 | 19.76 | 8.32 | 0.89 | 2.76 | 2.73 |
| Regional background | - | - | - | - | - | - | - | - | 3.87 | - | 163 | 55 | 89.7 | 24.9 | 2.68 | 20.8 |
