# Supplementary material for: Ecological risk assessment of migratory bird feces in plateau wetland: Phosphorus speciation, ARGs, and pathogens
Source: PLoS One. 2026 Sep 21;21(9):e0348709. doi: 10.1371/journal.pone.0348709 (PMC13592592; doi:10.1371/journal.pone.0348709)
Supplement: Supplementary file 2 [file pone.0348709.s002.docx]

**Table. S2 The detailed sequencing information of MBF.**

| Samples | Raw reads | Raw base (bp) | Clean reads | Clean base(bp) | Contigs | Contigs bases(bp) | N50(bp) | ORFs | Average Length(bp) |
| --- | --- | --- | --- | --- | --- | --- | --- | --- | --- |
| *G. nigricollis* | 49537938 | 7480228638 | 48763080 | 7301436548 | 314986 | 201373252 | 602 | 342287 | 435.74 |
| *G. grus* | 60200120 | 9090218120 | 59447398 | 8853907368 | 793191 | 634733808 | 926 | 1099560 | 509.51 |
| *F. atra* | 63232974 | 9548179074 | 62362790 | 9321409004 | 620821 | 490839189 | 898 | 824705 | 507.22 |
| *A. strepera* | 82820316 | 12505867716 | 80829232 | 12041976392 | 842399 | 536012978 | 634 | 1055301 | 443.36 |
| *A. zonorhyncha* | 55834116 | 8430951516 | 55037288 | 8198479331 | 737591 | 428703916 | 555 | 777717 | 413.28 |
| *M. penelope* | 52346892 | 7904380692 | 51431352 | 7682701410 | 773601 | 490888924 | 637 | 889933 | 440.42 |
| *T. ferruginea* | 76304692 | 11522008492 | 75252366 | 11158341737 | 1345608 | 748423223 | 551 | 1032748 | 342.69 |
